# An Antibody to the Lutheran Glycoprotein (Lu) Recognizing the LU4 Blood Type Variant Inhibits Cell Adhesion to Laminin α5

**DOI:** 10.1371/journal.pone.0023329

**Published:** 2011-08-12

**Authors:** Yamato Kikkawa, Takahiro Miwa, Yukiko Tohara, Takayuki Hamakubo, Motoyoshi Nomizu

**Affiliations:** Laboratory of Clinical Biochemistry, Tokyo University of Pharmacy and Life Sciences, Tokyo, Japan; Institut National de la Santé et de la Recherche Médicale (INSERM), France

## Abstract

**Background:**

The Lutheran blood group glycoprotein (Lu), an Ig superfamily (IgSF) transmembrane receptor, is also known as basal cell adhesion molecule (B-CAM). Lu/B-CAM is a specific receptor for laminin α5, a major component of basement membranes in various tissues. Previous reports have shown that Lu/B-CAM binding to laminin α5 contributes to sickle cell vaso-occlusion. However, as there are no useful tools such as function-blocking antibodies or drugs, it is unclear how epithelial and sickled red blood cells adhere to laminin α5 via Lu/B-CAM.

**Methodology/Principal Findings:**

In this study, we discovered a function-blocking antibody that inhibits Lu binding to laminin α5 using a unique binding assay on tissue sections. To characterize the function-blocking antibody, we identified the site on Lu/B-CAM recognized by this antibody. The extracellular domain of Lu/B-CAM contains five IgSF domains, D1-D2-D3-D4-D5. The antibody epitope was localized to D2, but not to the D3 domain containing the major part of the laminin α5 binding site. Furthermore, mutagenesis studies showed that Arg^175^, the LU4 blood group antigenic site, was crucial for forming the epitope and the antibody bound sufficiently close to sterically hinder the interaction with α5. Cell adhesion assay using the antibody also showed that Lu/B-CAM serves as a secondary receptor for the adhesion of carcinoma cells to laminin α5.

**Conclusion/Significance:**

This function-blocking antibody against Lu/B-CAM should be useful for not only investigating cell adhesion to laminin α5 but also for developing drugs to inhibit sickle cell vaso-occlusion.

## Introduction

The Lutheran glycoprotein (Lu) carries the antigen of the Lutheran blood group system. Lu is an Ig superfamily (IgSF) transmembrane protein in which the extracellular domain contains one variable, one constant-1 and three intermediate Ig-like domains, V-C1-I-I-I [1], [2], [3]. A splice variant of Lu known as basal cell adhesion molecule (B-CAM) [4] has the same extracellular and transmembrane domains as Lu, but it lacks the COOH-terminal 40 amino acids of the cytoplasmic tail. The cytoplasmic domain of Lu carries an SH3 binding motif, a dileucine motif and potential phosphorylation sites that could be involved in intracellular signaling pathways [1]. A dileucine motif at 608–609 mediates basolateral sorting of Lu in epithelial cells [5]. Protein kinase A phosphorylates Ser^621^ in the cytoplasmic tail and stimulates adhesion of sickled red blood cells to laminin under flow conditions [6]. Erythroid spectrin attaches to the Arg^573^Lys^574^ motif in the cytoplasmic tail of Lu and regulates its adhesive activity [7], [8]. Recent studies have shown that adrenergic stimuli increase Lu/B-CAM binding to laminin via both a PKA-dependent pathway and exchange proteins activated by the cAMP-dependent Rap1 pathway [9], [10]. However it is unclear if there is synergistic cross-talk between these pathways.

Lu/B-CAM binds with high affinity to laminin α5 but not to the other four laminin α chains [11], [12], [13], [14], [15]. Laminin α5 is widely expressed during development and in adult tissues and is the major α chain in many basement membranes [16]. The α5 chain associates with the γ1 chain and either β1 or β2 chains to form laminin-511 and -521, respectively [16]. Laminin-511/521 trimers are bound by several different receptors, including not only Lu/B-CAM but also integrins α3β1, α6β1, and α6β4 [17], [18] and α-dystroglycan [19]. These receptors bind to a laminin type globular (LG) domain found at the COOH-terminus of laminin α5 and consisting of five homologous subdomains in tandem (LG1–LG5) [19], [20]. α-dystroglycan binds primarily to the LG4–5 tandem [21], whereas the binding sites for Lu/B-CAM and α3β1/α6β1 integrins are localized on LG1–3 [20], [22]. Recently we showed that Lu/B-CAM and α3β1/α6β1 integrins bind competitively to the α5LG1–3 tandem [20].

Three groups have independently characterized the laminin α5 binding domain on Lu/B-CAM. One group showed that the fifth IgSF (D5) domain is critical for binding [23], whereas the other two groups localized the laminin α5 binding site to the first three IgSF domains (D1-D2-D3) [13], [24] and suggested that a specific spatial arrangement of D1-D2-D3 is required for the interaction. Recently, Mankelow et al reported the crystallographic structure of Lu/B-CAM using a combined approach of modeling and small angle X-ray scattering (SAXS) [2]. They found by site-directed mutagenesis that the laminin α5 binding site is formed from a patch of negatively-charged residues at the base of the D2 domain and the top of the D3 domain. Thus, it is likely that the binding site for laminin α5 is formed by the D2 and D3 domains. However, it remains a possibility that Lu/B-CAM could have two distinct binding sites for laminin α5.

The Lutheran glycoprotein (Lu) has been studied not only as the antigen of the Lutheran blood group system but also in the context of sickle cell disease. So far, several groups have shown that Lu/B-CAM binding to laminin α5 contributes to sickle cell vaso-occlusion [25], [26]. Therefore, development of a drug or antibody to interrupt this interaction has potential therapeutic implications [27]. A recent study showed that hydroxyurea reduced the adhesion of sickled red blood cells to laminin α5 via Lu [28]. On the other hands, there have been no antibodies to modulate the adhesion of sickled red blood cells to laminin α5.

Here we developed a soluble Lu binding assay using tissue sections. This allowed us to examine whether monoclonal antibodies to Lu/B-CAM could block its binding to laminin α5 in bona fide basement membranes. Of the tested commercial antibodies, we found that one monoclonal antibody against Lu/B-CAM could inhibit its binding to laminin α5. We hypothesized that the epitope of the function-blocking antibody might be near the laminin α5 binding region. To narrow the region on Lu/B-CAM recognized by this antibody, we produced a series of mutated recombinant Lu/B-CAM fragments. Furthermore, we found that the epitope contains an amino acid that comprises an antigenic site-related to Lu blood type. We also clarified the inhibitory mechanism whereby the antibody inhibited the binding of laminin α5 and examined the inhibitory effects of the antibody on the adhesion of hepatocellular carcinoma cells to the laminin α5 chain.

## Materials and Methods

### Proteins and antibodies

Recombinant proteins containing the Lu/B-CAM extracellular domain fused with a 6×His-Tag (Sol-Lu) or a Fc-Tag (Lu-Fc) were produced and characterized as described previously [15], [20]. Monoclonal antibodies against Lu/B-CAM are listed in Table 1. Rabbit antibody against domain LEb/L4a of mouse laminin α5 was a gift from Dr. Jeffrey Miner (Washington University School of Medicine, St Louis, MO). Polyclonal antibody against the globular (G) domain of laminin α2 was a gift from Dr. Peter D. Yurchenco (Robert Wood Johnson Medical School, Piscataway, NJ). Monoclonal antibodies against human integrin α1 (FB12), α3 (P1B5), α6 (GoH3) and β1 (6S6) were purchased from Millipore (Temecula, CA).

**Table 1:** Monoclonal antibodies to Lutheran/B-CAM.

Clone	Ig class	Function blocking	Antigen domain	Source	Reference
BRIC108	IgG1	−	D1	Biogenesis	Parsons et al., PNAS 1995, and Blood 1997
BRIC221	IgG2b	−	D4	SEROTEC	Parsons et al., PNAS 1995, and Blood 1997
87202	IgG1	−	D1	R&D system	-
87207	IgG2a	+	D2	R&D system	-

### Lu-Fc binding assay on tissue sections

Male C57BL/6J mouse (10–12 weeks old) were purchased from Nihon Charles River (Yokohama, Japan). Animal studies were permitted by Animal Research Committee of Tokyo University of Pharmacy and Life Sciences (Yaku 10–21). Mouse kidney and skeletal muscle were frozen whole by immersing in OCT compound and quick-freezing in 2-methylbutane cooled in a dry ice-ethanol bath. Sections were cut at 7 µm in a cryostat and air-dried. The sections were blocked in 10% normal goat serum. For Lu-Fc binding assays, Lu-Fc was adjusted to 1 µg/ml with Ca^2+^ and Mg^2+^ -free phosphate-buffered saline (PBS(−)) and placed on the sections. All incubations and washes were in PBS(−). Bound Lu-Fc was detected with anti-human IgG antibody conjugated to Alexa488 (Invitrogen, Carlsbad, CA, USA). Anti-laminin α5 polyclonal antibody was used for counter staining. Anti-rabbit IgG antibody conjugated to Alexa594 (Invitrogen, Carlsbad, CA) was used as secondary antibody. After several washes, sections were mounted in 90% glycerol containing 0.1× PBS(−) and 1 mg/ml *p*-phenylenediamine. Images were captured using Biozero (Keyence, Osaka, Japan). Fluorescence intensities of the bound recombinant proteins and laminin α5 were measured in the same areas and quantitated by using a BZ-analyzer (Keyence, Osaka, Japan). Fluorescence intensity of laminin α5 was used to normalize for differences in the amount of α5 chain in each section. For Lu-Fc inhibition assays, Lu-Fc was diluted with PBS(−) containing 10 µg/ml or 1∶10 dilution of anti-Lu/B-CAM monoclonal antibody. Immunocomplexes containing Lu-Fc and anti-Lu/B-CAM mAb were incubated on kidney sections, and Lu-Fc binding to laminin α5 was detected as described above.

### Construction of expression vectors

An expression vector containing deleted Lu/B-CAM fused with a Fc-tag was prepared as follows. The CD4-Ig vector was used as template for polymerase chain reaction (PCR) [29]. A DNA fragment encoding human IgG_1_ Fc was amplified using a specific primer set: 5′-GGAATTCCTTCTAGACGATCCCGAGGGTGAGTAGTACTAA-3′; 5′-TCCCTGTCTCCGGGTAAATGACCTAGG-3′. The PCR products were digested with EcoRI and AvrII and inserted into the EcoRI-XbaI sites of pcDNA3.1Neo (+). The full-length cDNAs for human Lu and melanoma cell adhesion molecule (Mel-CAM) were purchased from Invitrogen (Carlsbad, CA, USA) and used as template for PCR. cDNAs encoding the mutant proteins were generated by PCR using the primer sets listed Table S1 and inserted into the EcoRI and XbaI sites of the above human IgG1 Fc expression vector. For all PCR, KOD plus DNA polymerase (TOYOBO, Osaka, Japan) was used according to the manufacturer's instructions.

### Expression and purification of recombinant proteins

HEK293 cells were purchased from ATCC (Manassas, VA, USA) and maintained in DMEM containing 10% FBS. HEK293 cells were transfected with the deletion mutant Lu-Fc expression vectors using Lipofectamine 2000 (Invitrogen), and stable clones were selected using 400 µg/ml Zeocin (Invitrogen). All further cell culture was carried out in the presence of the antibiotic. The selected cells were grown to confluency in culture dishes with DMEM containing 10% FBS. The confluent cells were incubated in serum free DMEM for 4 days. The conditioned media were harvested and clarified by sequential centrifugation at 500 rpm for 10 min and 10,000 rpm for 20 min. The collected media were precipitated with ammonium sulfate at 80% saturation. The resulting precipitates were collected by centrifugation at 10,000 rpm for 30 min, and then dissolved in and dialyzed against PBS(−). The 30-fold concentrated media were used for purification. The recombinant proteins were purified from the conditioned culture media by Protein A sepharose (GE Health Care Bio-Science, Piscataway, NJ, USA). The eluted fractions were pooled and dialyzed against PBS(−). The purified proteins were separated by SDS-PAGE using 7.5% gels under reducing conditions. The separated proteins were stained with Coomassie Brilliant Blue.

### Antigen capture ELISA

ELISA plates were coated with 3 µg/ml of Sol-Lu and blocked with 1% bovine serum albumin (BSA) in PBS(−). Diluted monoclonal antibodies were incubated with recombinant proteins for 1 hour at room temperature and then transferred into the Sol-Lu-coated wells. After a further 1 hour incubation and washing with PBS(−) containing 0.05% Tween 20, bound antibodies were detected by addition of horseradish-peroxidase conjugated anti-mouse IgG (GE Healthcare, Piscataway, NJ, USA), followed by addition of 0.4 mg/ml *o*-phenylendiamine and 0.01% H_2_O_2_. The optical density at 450 nm was measured in a model 550 microplate reader (Bio-Rad Laboratories, Hercules, CA, USA).

### Cell adhesion inhibition assays

Human hepatocellular carcinoma cell line HuH-7 was purchased from Health Science Research Resources Bank (Osaka, Japan) and maintained in DMEM containing 10% FBS. Cell adhesion assays were performed as described previously [30]. Briefly, 96-well microtiter plates (Nunc, Roskilde, Denmark) were incubated with 2.5 µg/ml of recombinant laminin-511 at 37°C for 1 h, and then blocked with PBS(−) containing 1% BSA for another hour. HuH-7 cells were suspended in serum-free DMEM at a density of 4×10^5^ cells/ml and preincubated with 1 µg/ml of antibody at room temperature for 10 min. 50 µl of cell suspension were added to wells coated with recombinant laminin-511. After incubation at 37°C for 1 h, the attached cells were fixed with 4% formaldehyde, stained with Diff-Quik (International Regents Corp., Kobe, Japan), and counted under the microscope.

## Results

### Inhibitory effects of monoclonal antibodies on the binding of Lu-Fc to laminin α5 in mouse tissue sections

**Figure 1:**
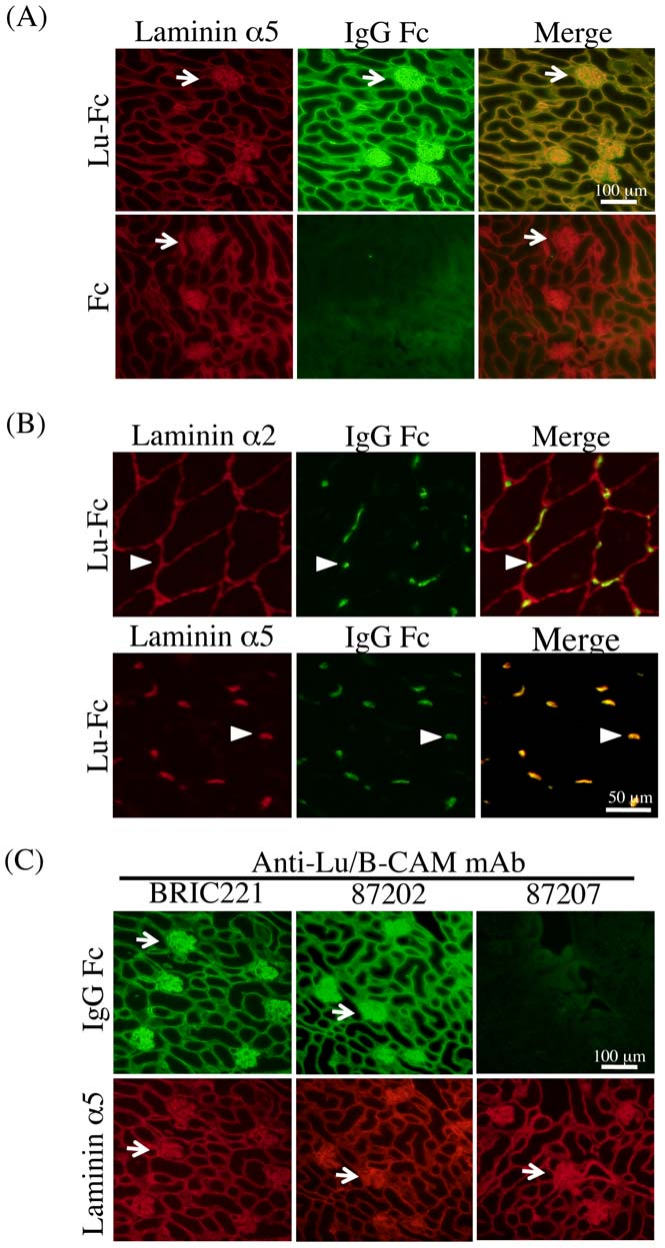
Lu/B-CAM binding assay on tissue sections. (A) Binding of Lu-Fc to laminin α5 in adult mouse kidney sections. Sections were incubated with Lu-Fc (upper panel) or Fc (lower panel) at room temperature for 1 hour. Endogenous laminin α5 and bound Lu-Fc were detected with anti-laminin α5 domain LEb/L4b and anti-human IgG, as indicated. Merged pictures showed that laminin α5 and Lu-Fc co-localized at basement membranes. Arrows indicate glomeruli, laminin α5-rich structures. Bar, 100 µm. (B) Binding of Lu-Fc to laminin α5 in adult mouse skeletal muscle sections. Endogenous laminin α2 (upper panel) and α5 (lower panel) were detected with antisera. Bound Lu-Fc was detected with an antibody against human IgG as described above. Lu-Fc only bound to the basement membranes containing α5, primarily neuromuscular junctions. Arrowheads indicate neuromuscular junctions. Bar, 50 µm. (C) Inhibitory effects of anti-Lu/B-CAM antibodies on Lu-Fc binding to laminin α5. After preincubation with anti-Lu/B-CAM antibodies, Lu-Fc was used in the binding assay on tissue sections. Antibody 87207 inhibited the binding of Lu-Fc to endogenous laminin α5.

In a previous study we prepared a dimerized soluble recombinant protein (Lu-Fc) composed of the Lu extracellular domain and human IgG_1_ Fc region [20]. Lu-Fc dimerizes via the Fc region and should bind to laminin α5 more effectively than the monomeric Lu (Sol-Lu) used in our earlier study [15]. In addition, although Sol-Lu worked effectively as a histochemical probe on fetal tissue sections [15], the need to use a mouse monoclonal antibody to detect it restricts its usefulness on adult mouse tissues containing endogenous immunoglobulins. Therefore, here we used Lu-Fc as a probe on adult mouse tissue sections because it could be detected by anti-human IgG antibody conjugated to Alexa488. Lu-Fc, but not Fc alone, bound essentially all basement membranes containing laminin α5 in the adult mouse kidney tissues (Fig. 1A). In adult mouse skeletal muscle basement membranes, laminin α5 is found primarily at neuromuscular junctions [31]. Lu-Fc bound to neuromuscular junctions but not to the extrasynaptic basement membrane, suggesting that Lu/B-CAM is a specific receptor for α5 (Fig. 1B). The binding of Lu-Fc to laminin α5 in tissue sections also allowed us to examine whether anti-Lu/B-CAM monoclonal antibodies could block binding (Fig. 1C). Before incubation on kidney sections, Lu-Fc was incubated with anti-Lu/B-CAM monoclonal antibodies. Antibody 87207 inhibited binding to laminin α5, but BRIC221, 87202, and BRIC108 antibodies did not (Fig. 1C and Table 1).

### Epitope mapping of antibodies on Lu/B-CAM

**Figure 2:**
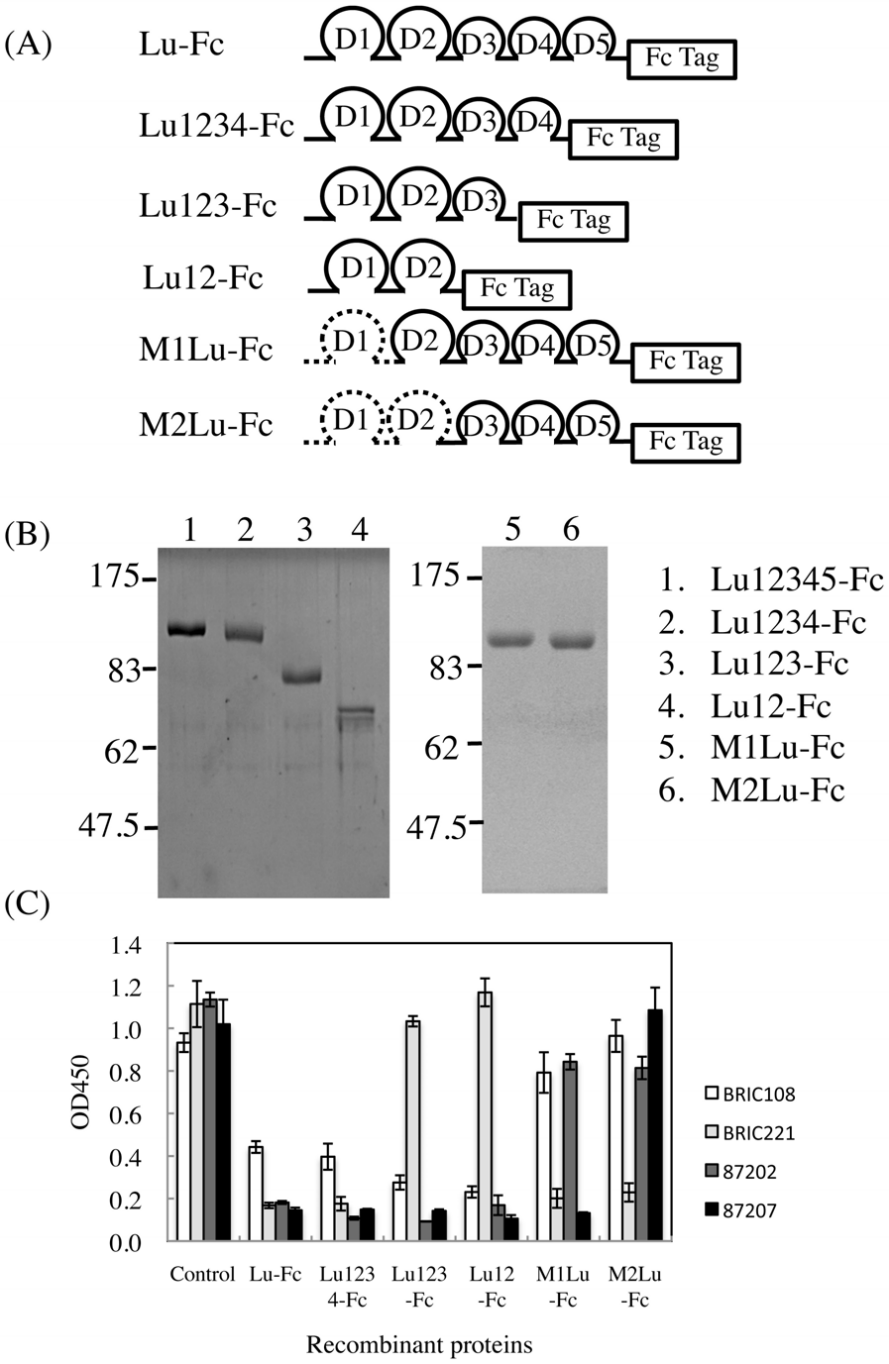
Identifying the IgSF domains recognized by anti-Lu/B-CAM antibodies. (A) Diagram of the deletion and chimeric mutant proteins designed to narrow the epitopes of anti-Lu/B-CAM antibodies. For chimeric mutant proteins, the D1 or D1–D2 domains of Lu-Fc were replaced with the analogous domains of melanoma cell adhesion molecule (Mel-CAM). The deletion and chimeric mutant proteins were fused with an IgG Fc domain tag. (B) The mutant proteins purified from conditioned media of HEK293 transfectants were subjected to SDS-PAGE on a 7.5% gel under reducing conditions. Protein was stained with Coomassie Brilliant Blue. Molecular mass standards are indicated. (C) ELISA using the indicated anti-Lu/B-CAM antibodies absorbed with the various recombinant proteins. Wells were coated with 3 µg/ml of Sol-Lu. The mutant proteins were mixed with the diluted antibodies at 1 µg/ml. Each bar represents the mean of triplicate assays. Error bars indicate standard deviation.

**Figure 3:**
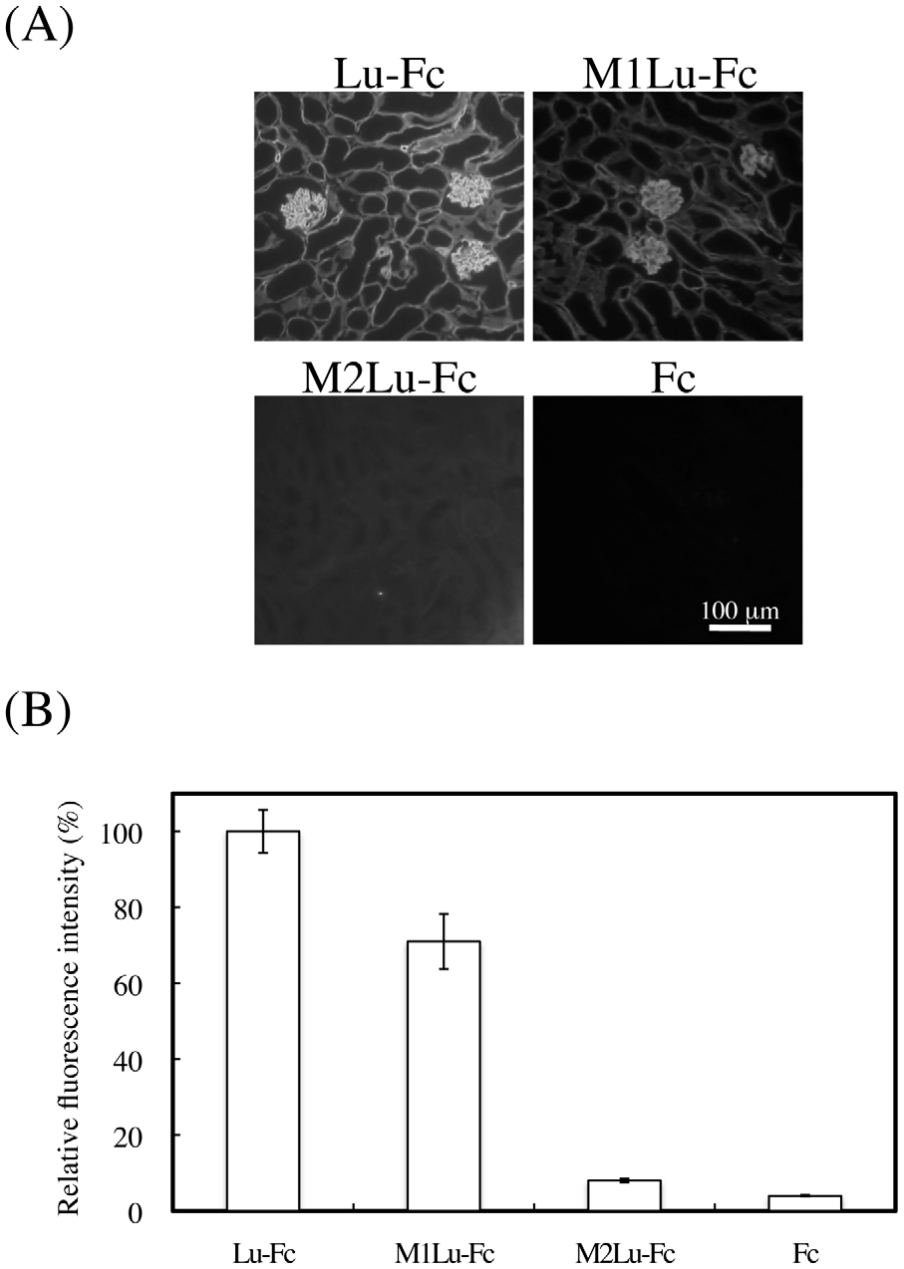
The binding of Lu-Fc and chimeric mutant proteins to laminin α5 in tissue sections. (A) Sections of adult mouse kidney were incubated with the Lu-Fc, and chimeric mutants, as indicated. The recombinant proteins bound to endogenous laminin α5 were detected with an antibody against human IgG. Bar, 100 µm. M1Lu-Fc bound to laminin α5 but not M2Lu-Fc did. (B) Quantitation of the recombinant proteins bound to laminin α5. Fluorescence intensities were measured as described in Materials and Methods. The binding of Lu-Fc was set to 100. Each bar represents the mean of triplicate assays. Error bars indicate standard deviation.

It is possible that the function-blocking antibody 87207 recognized an epitope of Lu/B-CAM involved in binding laminin α5. To map the epitopes of Lu/B-CAM antibodies, Lu-Fc deletion and chimeric mutant proteins (Fig. 2A) were produced in HEK293 cells. The recombinant proteins were purified from the culture media using Protein A Sepharose. The purified proteins were subjected to SDS-PAGE under reducing conditions (Fig. 2B). The mutant proteins migrated at molecular weights predicted from their cDNA sequences. We also tried to produce a deletion mutant protein composed of the D1 domain. This deletion mutant protein did not react with the antibody recognizing the D1 domain (BRIC108), suggesting that it might be misfolded. Therefore we produced chimeric mutant proteins in which the D1 or D1–D2 tandem was replaced with the analogous domain of melanoma cell adhesion molecule (Mel-CAM). Epitope mapping of BRIC108, BRIC221, and 87202, which did not inhibit the binding of Lu to laminin α5, was performed to examine whether the mutant proteins were properly folded. The antibodies were diluted with washing buffer containing the purified proteins. After incubation for 1 hour, the diluted antibodies were transferred into the wells coated with Sol-Lu. When the four antibodies were adsorbed to either Lu-Fc or Lu1234-Fc, they did not react with the Sol-Lu-coated wells (Fig. 2C), indicating that they bound the purified proteins. Lu-Fc, Lu1234-Fc, Lu123-Fc and Lu12-Fc, but not M1Lu-Fc and M2Lu-Fc, bound the BRIC108 antibody, which reacts with the D1 domain (Fig. 2C). Although Lu123-Fc and Lu12-Fc were bound by BRIC108, 87202 and 87207, BRIC221 did not react with it but instead bound to the Sol-Lu-coated wells. As shown in previous study [13], the epitope of BRIC221 was localized to the D4 domain. The 87202 antibody did not react with M1Lu-Fc, indicating that the epitope of the antibody is localized in D1, similar to BRIC108. The antibody 87207, which inhibited the binding of Lu to laminin α5, reacted with M1Lu-Fc but not M2Lu-Fc, showing its epitope to be in D2 (Fig. 2C). We also examined whether the deletion and chimeric mutant proteins bound to laminin α5 in tissue sections. Although data are not shown, the deletion mutant proteins Lu1234-Fc and Lu123-Fc exhibited binding activity on laminin α5 but Lu12-Fc did not. On the other hand, M1Lu-Fc bound to laminin α5, but M2Lu-Fc did not (Fig. 3). These results strongly suggest that the D2 domain is involved in the binding of laminin α5.

### Analysis of Lu-Fc mutants with reduced laminin α5 interaction

**Figure 4:**
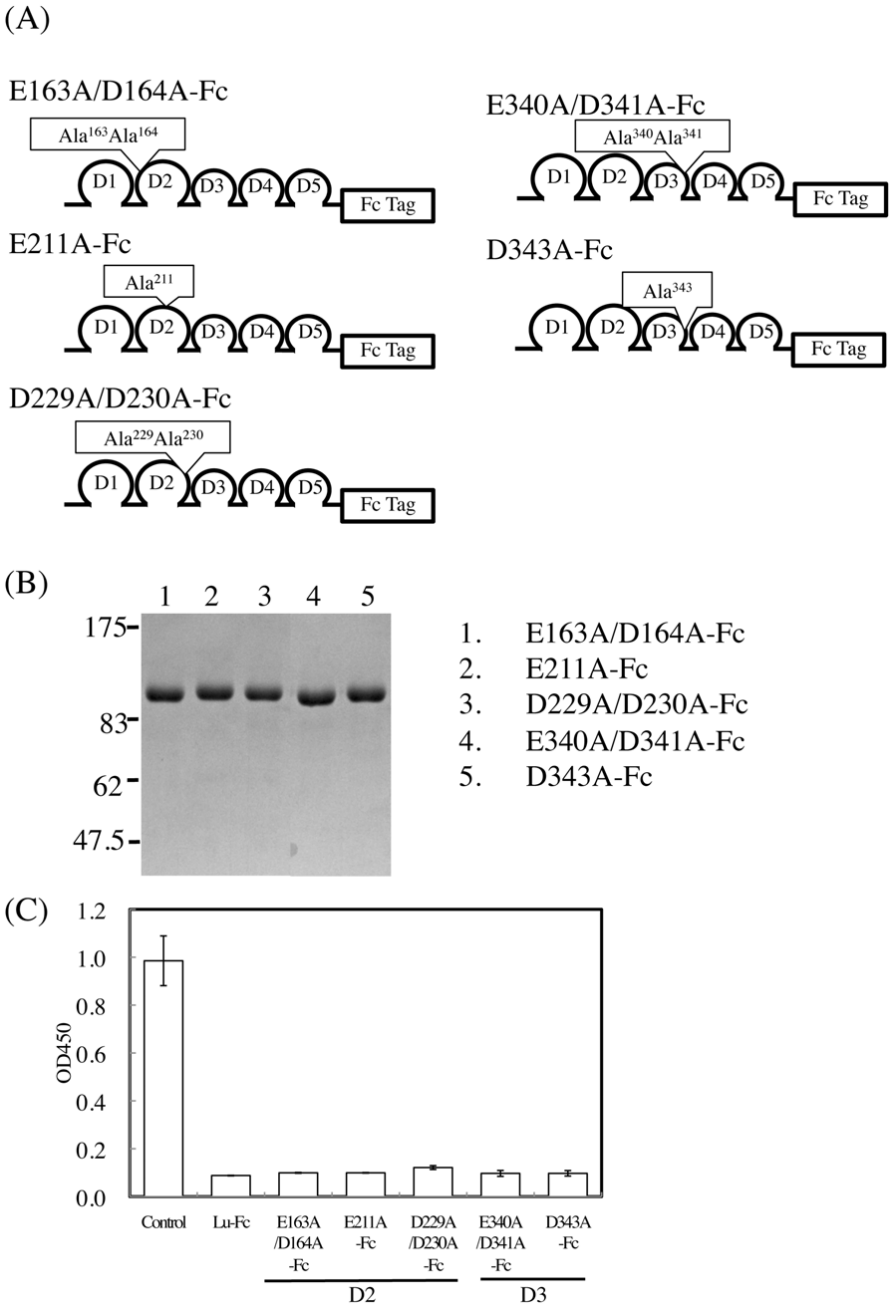
Lu-Fc mutants with reduced interaction with laminin α5 still bind antibody 87207. (A) Diagrams of Lu-Fc variants mutated at acidic residues involved in laminin α5 binding. Glu^163^Asp^164^, Glu^211^, and Asp^229^Asp^230^ were localized in the D2 domain, and Glu^340^, Asp^341^, and Asp^343^ in the D3 domain. Each of these amino acids was substituted with Ala. (B) The purified mutant proteins were subjected to SDS-PAGE on a 7.5% gel under reducing conditions and stained with Coomassie Brilliant Blue. Molecular mass standards are indicated. (C) ELISA using 87207 absorbed with the mutant recombinant proteins before addition to the wells of microtiter plates coated with Sol-Lu. Each bar represents the mean of triplicate assays. Error bars indicate standard deviation.

Recently, Mankelow et al. reported that a cluster of negatively charged residues in the D2 and D3 domains forms the laminin α5-binding site [2]. Therefore, these negatively charged residues may be part of the epitope of antibody 87207, which inhibited binding to laminin α5. Lu-Fc mutants with one or two of these charged amino acids substituted with alanine were produced in HEK293 cells (Fig. 4A). The purified recombinant proteins were subjected to SDS-PAGE under reducing conditions (Fig. 4B). When 87207 was adsorbed to these Lu-Fc mutants, it could not bind to Sol-Lu-coated wells (Fig. 4C). 87207 did not recognize these acidic residues in the D2 and D3 domains. These results suggest that 87207 binding to its epitope covers and masks the acidic amino acid residues contributing to the interaction of Lu/B-CAM with laminin α5.

### Fine mapping of the 87207's epitope on the D2 domain of Lu/B-CAM

**Figure 5:**
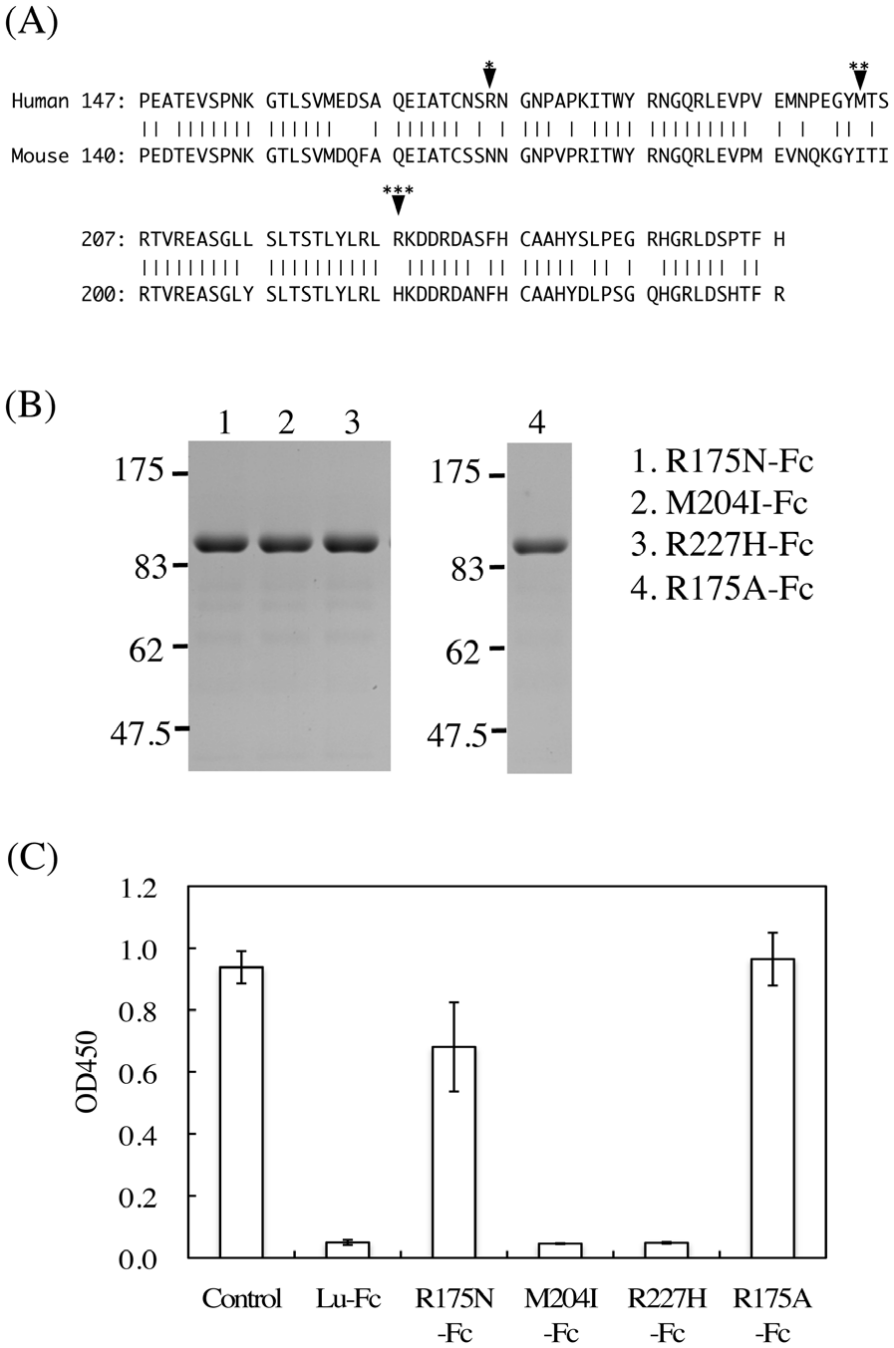
Fine mapping of the epitope recognized by 87207. (A) Alignment of the human and mouse Lutheran D2 domain sequences. Vertical lines connect amino acids that are identical across species. Arrowheads represent variant Lutheran antigenic sites in the human sequence (*: LU4/LU-4, Arg175Gln; **: LU8/LU14, Met204Lys; ***: LU16/LU-16, Arg227Cys). Arg^175^, Met^204^, and Arg^227^ in Lu-Fc were substituted with Asn (or Ala), Ile, and His, the corresponding mouse residues, respectively. (B) The purified mutant proteins were subjected to SDS-PAGE on a 7.5% gel under reducing conditions and stained with Coomassie Brilliant Blue. Molecular mass standards are indicated. (C) ELISA using 87207 absorbed with the mutant recombinant proteins before addition to the wells of microtiter plates coated with Sol-Lu. Each bar represents the mean of triplicate assays. Error bars indicate standard deviation. Antibody 87207 did not react with R175N-Fc and R175A-Fc, indicating that Arg^175^ is required for immunoreactivity.

We sought to more finely identify the epitope of 87207 on the D2 domain of Lu/B-CAM. The antibody 87207 was produced in a mouse immunized with human Lutheran. Therefore, the differences between the human and mouse Lu/B-CAM amino acid sequences (Fig. 5A) should be responsible for its immunogenicity. Lutheran, as a determinant of the blood group system, consists of 19 documented antigens [27] deriving from changes in amino acid residues that are likely exposed on the surface of Lu/B-CAM. Three of these antigenic sites, Arg^175^, Met^204^, and Arg^227^, are localized in the D2 domain. These amino acid residues also differ from the corresponding mouse Lu/B-CAM residues. We considered the possibility that these amino acid residues could be part of the 87207 epitope. To test this, Arg^175^, Met^204^, and Arg^227^ were individually substituted with the corresponding amino acids found in mouse Lu/B-CAM: Asn^175^, Ile^204^, and His^227^, respectively. The mutated recombinant proteins fused with the Fc tag were generated by site-directed mutagenesis. The recombinant proteins were purified from the culture media using Protein A Sepharose and subjected to SDS-PAGE under reducing conditions, which showed the appropriate size (Fig. 5B). Preincubation with 87207 showed that although the antibody reacted with M204I-Fc and R227H-Fc, R175N-Fc was not immunoreactive (Fig. 5C). Moreover, to confirm if Arg^175^ was essential for forming the epitope, we produced mutant protein substituted with Ala. R175A-Fc also lacked the immunoreactivity of 87207. This indicates that Arg^175^ is critical for forming the 87207 epitope.

### The mechanism of 87207-mediated inhibition of Lu/B-CAM binding to laminin α5

**Figure 6:**
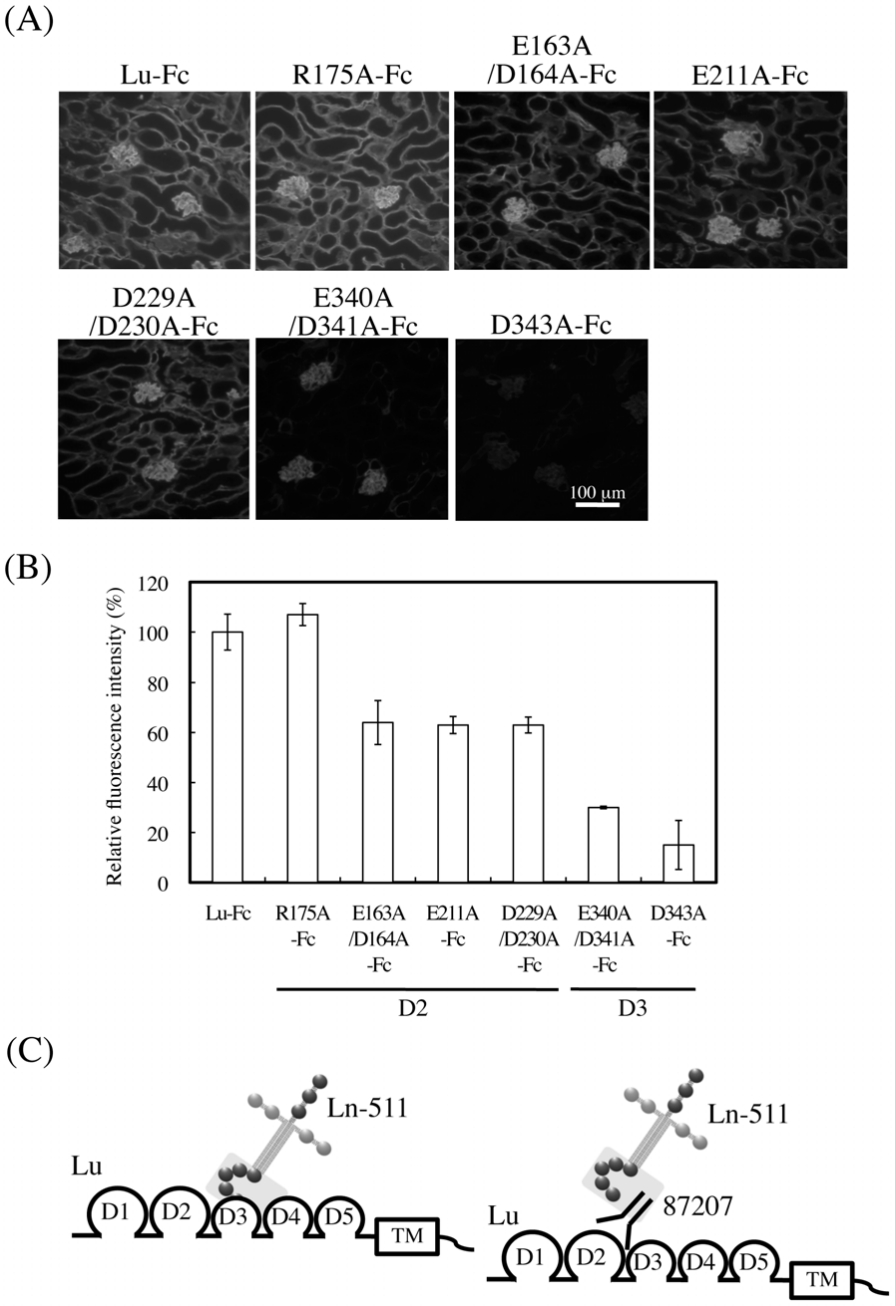
Analysis of Lu-Fc mutant binding to laminin α5 in tissue sections. (A) Sections of adult mouse kidney were incubated with the Lu-Fc, and Lu-Fc mutants, as indicated. The recombinant proteins bound to endogenous laminin α5 were detected with an antibody against human IgG. Bar, 100 µm. (B) Quantitation of the recombinant proteins bound to laminin α5. Fluorescence intensities were measured as described in Materials and Methods. The binding of Lu-Fc was set to 100. Each bar represents the mean of triplicate assays. Error bars indicate standard deviation. In comparison with wild type, the mutations in the D2 domain reduced the interaction with laminin α5. The binding of E340A/D341A-Fc and D343A-Fc to endogenous laminin α5 was mostly abolished. (C) Schema of the inhibitory effects of antibody 87207 on the binding of Lutheran to laminin α5. The D3 domain, rather than the D2 domain, is most important for α5 binding. Antibody 87207 recognizes the D2 domain but spatially inhibits the binding of laminin α5 to the D3 domain that is adjacent.

To examine if Arg^175^ was involved in the binding of laminin α5, the binding assay on mouse kidney tissue sections was performed using R175A-Fc (Fig. 6A). Fluorescence intensity was quantitated to measure the binding of the mutant proteins to laminin α5 (Fig. 6B). Although Arg^175^ was a part of the epitope recognized by the function-blocking antibody, it was not involved in the binding of laminin α5. Furthermore, to clarify a mechanism whereby 87207 inhibits the binding of Lu to laminin α5, the binding of mutant proteins was examined on mouse kidney tissue sections. Lu-Fc containing the mutations in the D2 domain bound to endogenous laminin α5 on mouse kidney sections (Fig. 6A), but they reduced the interaction with laminin α5 (Fig. 6B). Furthermore, E340A/D341A-Fc and D343A-Fc (mutations in the D3 domain) exhibited little or no binding activity (Fig. 6A and B). As shown in Fig. 3, M2Lu-Fc, for which the D1–D2 tandem of Lu was replaced with the analogous tandem of Mel-CAM, lost the epitope of the function-blocking antibody and did not bind to laminin α5. The straightforward interpretation was that the D2 domain was involved in the binding of laminin α5. However, our mutagenesis study also showed the D3 domain to be a major region for laminin α5 binding, as described in Mankelow et al [2]. These results suggest that the D2 domain is essential for formation of the binding site but not the interaction with the laminin α5 chain. Therefore, we conclude that, because the major α5 binding sites are localized in the D3 but not the D2 domain, antibody 87207 seems to sterically hinder Lu/B-CAM binding to laminin α5 (Fig. 6C).

### Inhibitory effects of 87207 on adhesion of HuH-7 cells to laminin α5

**Figure 7:**
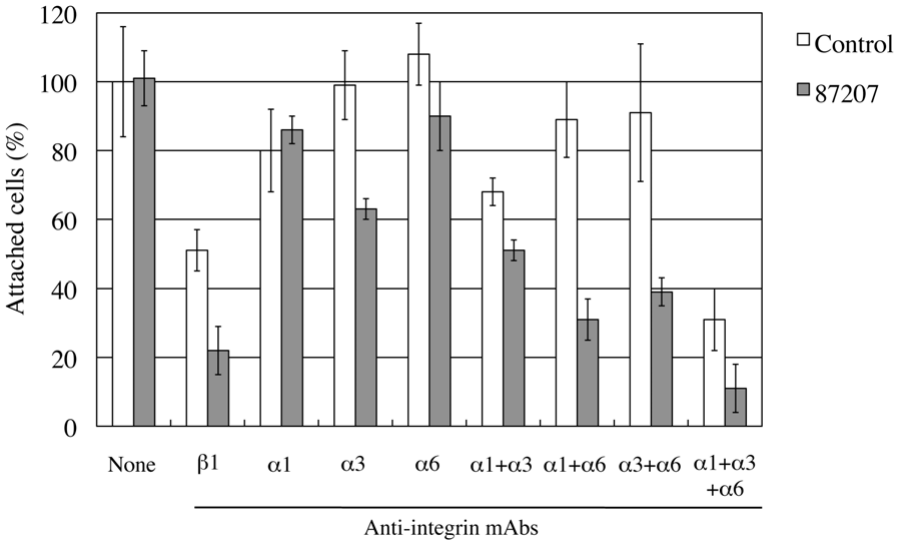
Inhibitory effects of antibody 87207 on adhesion of HuH-7 cells to laminin α5. HuH-7 cells pre-incubated with antibody 87207 and function-blocking antibodies against the indicated integrin subunits were added to laminin-511-coated wells. After incubation for 1 hr, the attached cells were stained and counted. Values are expressed as percentages of the number of cells adhering in the absence of antibody. Each column represents the mean of triplicate assays. Bars, standard deviation. Antibody 87207 alone had no effect, but cooperated with anti-integrin β1 or a combination of the three integrin α chain antibodies to inhibit adhesion by ∼90%.

To examine if antibody 87207 can inhibit Lu/B-CAM-mediated cell adhesion to laminin α5 in vitro, cell adhesion assays were performed using HuH-7 cells derived from a human hepatocellular carcinoma. HuH-7 cells express α1β1/α3β1/α6β1 integrins and Lu/B-CAM that can bind to laminin-511 [30]. After incubation with antibodies, the cells were plated on dishes coated with recombinant laminin-511 (α5β1γ1). Although antibody 87207 alone had no effect on adhesion of HuH-7 cells to laminin-511 (Fig. 7), the remaining adhesion of HuH-7 cells to laminin α5 in the presence of an inhibitory anti-integrin β1 antibody was significantly reduced by addition of antibody 87207. Moreover, combining antibody 87207 with inhibitory antibodies to α1/α3/α6 integrins dramatically reduced adhesion of HuH-7 cells to laminin-511.

## Discussion

Soluble receptor binding assays on tissues is a proven method to reveal the presence of ligands [32]. To visualize and localize soluble receptors bound to ligands, they have been conjugated with alkaline phosphatase or detected with a specific monoclonal antibody [15], [32]. In the present study, we developed a binding assay on tissue sections using Lu-Fc, consisting of the ectodomain of Lu/B-CAM fused with a human Fc-tag. The staining of laminin α5 and Lu-Fc overlapped, indicating that Lu-Fc bound to endogenous laminin α5 in tissue sections. Therefore, it is unlikely that an unknown ligand for Lu/B-CAM exists in adult mouse kidney and skeletal muscle. The use of a Fc-tagged soluble receptor in binding assays on tissue sections was advantageous, as Lu-Fc was easily purified by Protein A Sepharose and detected with anti-human IgG antibody conjugated to Alexa488. Fc-tagged soluble receptors should be useful tools to identify unknown ligands in adult tissue sections.

The binding assays also allowed us to characterize monoclonal antibodies to Lu/B-CAM. The results showed that antibody 87207 inhibited the binding of Lu-Fc to laminin α5. The adhesion assays also showed that combining antibody 87207 with anti-integrin antibodies inhibited adhesion of hepatocellular carcinoma cells to laminin-511. In our previous studies, recombinant proteins containing Lu extracellular domains were used to prevent the binding of Lu/B-CAM to laminin α5 [20]. However, since Lu/-CAM and α3β1/α6β1 integrins bind competitively to the LG1–3 tandem of laminin α5, the possibility that they inhibited the binding of integrins to laminin α5 was excluded. Because antibody 87207 directly inhibited the binding of Lu/B-CAM to laminin α5, it could be a useful tool to clarify the role of Lu/B-CAM in cell-adhesion to laminin α5. In future experiments it will be crucial to examine whether 87207 inhibits the adhesion of human sickle cells to laminin α5. Our results also showed that cell adhesion to laminin α5 was not inhibited in the presence of Lu antibody alone. In a previous study, we showed that Lu and integrins competitively bind to the LG1–3 tandem of laminin α5 [20]. When Lu binding was inhibited by the 87207 antibody, free integrins on the surface of HuH-7 cells were apparently able to interact with the LG1–3 tandem and mediate binding. On the other hand, Lu binding could not compensate for inhibition by anti-integrin antibodies, which could block cell adhesion to laminin α5. Therefore we conclude that Lu serves as a secondary receptor for laminin α5 in adhesion of HuH-7 cells.

Recently, the laminin α5 binding site on Lu/B-CAM was identified using x-ray crystallography, small-angle x-ray scattering, and site-direct mutagenesis [2]. Mutagenesis studies indicated that negatively charged residues in the D2 and D3 domain form the laminin α5 binding site; of them, acidic residues in the D3 domain are the primary contributors. These results made us hypothesize that the function-blocking antibody directly recognized the binding site of laminin α5 in the D3 domain. However the epitope recognized by the function-blocking antibody was localized to the D2 domain. Furthermore, M2Lu-Fc lacking the D2 domain of Lu lost binding activity, and 87207 completely abolished Lu binding to laminin α5. These results led us to consider that the D2 domain was the major part of the α5 binding site. Therefore we examined whether Glu^163^, Asp^164^, Glu^211^, Asp^229^, and Asp^230^ in the D2 domain, which contribute to the interaction with laminin α5, were immunoreactive with 87207. However these acidic amino acid residues did not form the epitope of 87207. Instead, antibody 87207 may cover the laminin α5 binding site on the D2 domain of Lu/B-CAM. On the other hand, our binding assay on tissue sections showed that Glu^340^, Asp^341^, and Asp^343^ in the D3 domain was the major part of the α5 binding site, as described in Mankelow et al [2]. As shown in Fig. 5C, the inhibitory mechanism of 87207 seems to involve steric hindrance of Lu/B-CAM binding to laminin α5. The D2 domain may also be essential for exposing the binding site of laminin α5 on the D3 domain rather than directly interacting with α5. The Lu/B-CAM model predicts a rod-like structure with a flexible hinge region of 8 residues between the D2 and D3 domains. Mutagenesis studies show that a small deletion in the hinge region also abolished the binding to laminin α5 [2], suggesting that the flexible junction is essential for ligand binding. Although the epitope was not localized adjacent to this hinge region, the bound antibody may impact its flexibility.

Lu has been studied as the antigen of the Lutheran blood group system, which consists of 19 antigens numbered from LU1 to LU21, with two of these declared obsolete [27]. 87207 recognized the D2 domain, which contains the antigens for human alloantibodies LU4, LU8, LU14, and LU16. We suspected that the amino acids relevant to these determinants would be exposed on the surface of human Lu/B-CAM and would be particularly immunogenic in mice. In fact, none of these amino acids is conserved in mouse Lu/B-CAM. Our mutagenesis studies showed that Arg^175^, the site of LU4/LU-4, was a pivotal amino acid in the epitope recognized by the function-blocking antibody 87207. We also examined whether Arg^175^ contributed to laminin α5 binding. As shown in Fig. 5B, substitution with Ala did not affect the binding of laminin α5 to Lu/B-CAM. Arg^175^ seemed to be only a part of the epitope. The epitope recognized by a typical monoclonal antibody is formed by several residues. In future experiments we will identify the other residues required for formation of the epitope recognized by 87207.

Lu has also been studied in the context of sickle cell disease [27], [33]. The increased adhesion of sickled red cells to laminin α5 via Lutheran binding is suspected to contribute to vaso-occlusion in patients [25], [26]. To prove this hypothesis, the generation of inhibitory antibodies or small molecules is required. Here we showed that a monoclonal antibody can inhibit the binding of Lu to laminin α5. Although the epitope of 87207 was not part of the laminin α5 binding site, this epitope may still be useful for developing drugs and humanized antibodies to inhibit the vaso-occlusion common in sickle cell disease.

## Supporting Information

Table S1 **Specific primer sets for mutant proteins are listed in Table S1.**
(DOC)
