# Supplementary material for: An Antibody to the Lutheran Glycoprotein (Lu) Recognizing the LU4 Blood Type Variant Inhibits Cell Adhesion to Laminin α5
Source: PLoS One. 2011 Aug 12;6(8):e23329. doi: 10.1371/journal.pone.0023329 (PMC3155534; doi:10.1371/journal.pone.0023329)
Supplement: Table S1 — Specific primer sets for mutant proteins are listed in Table S1. (DOC) [file pone.0023329.s001.doc]

| Table S1 Primer sets | | |
| --- | --- | --- |
| rProtein | Primer | Sequence (5'-3') |
| Lu1234-Fc | HLU03 | GGAATTCGCCACCATGGAGCCCCCGGACGCACCG |
|  | HLU70 | CGTCTAGACCCGAGCCTTGGACCAGCAGCGTGAA |
| Lu123-Fc | HLU03 | GGAATTCGCCACCATGGAGCCCCCGGACGCACCG |
|  | HLU68 | CGTCTAGACCGTCCAGATAGGCCACGCGCAGCTC |
| Lu12-Fc | HLU03 | GGAATTCGCCACCATGGAGCCCCCGGACGCACCG |
|  | HLU66 | CGTCTAGACCGCTGCCCACCCAGAACTGCACGTG |
| M1Lu-Fc | MCAM01 | GGAATTCGCCACCATGGGGCTTCCCAGGCTGGTC |
|  | MCAM10 | GGCCTCTGGCTTTGCAAACACCTGGATGCGGTACTCCTGGGA |
|  | HLU65 | TCCCAGGAGTACCGCATCCAGGTGTTTGCAAAGCCAGAGGCC |
|  | HLU34 | CGTCTAGACCCACTCCAGCCTGGGAGGTCTG |
| M2Lu-Fc | MCAM01 | GGAATTCGCCACCATGGGGCTTCCCAGGCTGGTC |
|  | MCAM12 | CGTGGGATAGTGCAGGGTGAGGGTGACTTCCCTGGACTCCTT |
|  | HLU75 | AAGGAGTCCAGGGAAGTCACCCTCACCCTGCACTATCCCACG |
|  | HLU34 | CGTCTAGACCCACTCCAGCCTGGGAGGTCTG |
| E163A/D164A-Fc | HLU03 | GGAATTCGCCACCATGGAGCCCCCGGACGCACCG |
|  | HLU112 | GATCTCCTGGGCAGAGGCCGCCATCACAGACAGTGTCCCTTT |
|  | HLU83 | ACACTGTCTGTGATGGCGGCCTCTGCCCAGGAGATCGCCACC |
|  | HLU34 | CGTCTAGACCCACTCCAGCCTGGGAGGTCTG |
| E211A-Fc | HLU03 | GGAATTCGCCACCATGGAGCCCCCGGACGCACCG |
|  | HLU114 | GGAGAGCAGGCCCGAGGCCGCCCGGACCGTGCGGCTGGTCAT |
|  | HLU85 | ACCAGCCGCACGGTCCGGGCGGCCTCGGGCCTGCTCTCCCTC |
|  | HLU34 | CGTCTAGACCCACTCCAGCCTGGGAGGTCTG |
| D229A/D230A-Fc | HLU03 | GGAATTCGCCACCATGGAGCCCCCGGACGCACCG |
|  | HLU116 | GAAGCTGGCGTCTCGGGCCGCCTTGCGGAGCCGCAGGTAGAG |
|  | HLU87 | CTGCGGCTCCGCAAGGCGGCCCGAGACGCCAGCTTCCACTGC |
|  | HLU34 | CGTCTAGACCCACTCCAGCCTGGGAGGTCTG |
| E340A/D341A-Fc | HLU03 | GGAATTCGCCACCATGGAGCCCCCGGACGCACCG |
|  | HLU120 | ATCTGCCGCGTCGTAAGCCGCCACTCTGCAGCCATAGGTCCC |
|  | HLU91 | TATGGCTGCAGAGTGGCGGCTTACGACGCGGCAGATGACGTG |
|  | HLU34 | CGTCTAGACCCACTCCAGCCTGGGAGGTCTG |
| D343A-Fc | HLU03 | GGAATTCGCCACCATGGAGCCCCCGGACGCACCG |
|  | HLU118 | CTGCACGTCATCTGCCGCGGCGTAATCCTCCACTCTGCAGCC |
|  | HLU89 | TGCAGAGTGGAGGATTACGCCGCGGCAGATGACGTGCAGCTC |
|  | HLU34 | CGTCTAGACCCACTCCAGCCTGGGAGGTCTG |
| R175N-Fc | HLU03 | GGAATTCGCCACCATGGAGCCCCCGGACGCACCG |
|  | HLU96 | GTTCCCGTTGTTGCTGTTGCAGGTGGCGATCTC |
|  | HLU55 | TGCAACAGCAACAACGGGAACCCGGCCCCCAAG |
|  | HLU34 | CGTCTAGACCCACTCCAGCCTGGGAGGTCTG |
| M204I-Fc | HLU03 | GGAATTCGCCACCATGGAGCCCCCGGACGCACCG |
|  | HLU98 | GCGGCTGGTTATGTAGCCCTCTGGGTTCATCTC |
|  | HLU57 | GAGGGCTACATAACCAGCCGCACGGTCCGGGAG |
|  | HLU34 | CGTCTAGACCCACTCCAGCCTGGGAGGTCTG |
| R227H-Fc | HLU03 | GGAATTCGCCACCATGGAGCCCCCGGACGCACCG |
|  | HLU94 | GTCATCCTTGTGGAGCCGCAGGTAGAGGGTGCT |
|  | HLU53 | CTGCGGCTCCACAAGGATGACCGAGACGCCAGC |
|  | HLU34 | CGTCTAGACCCACTCCAGCCTGGGAGGTCTG |
| R175A-Fc | HLU03 | GGAATTCGCCACCATGGAGCCCCCGGACGCACCG |
|  | HLU106 | GTTCCCGTTCGCGCTGTTGCAGGTGGCGATCTC |
|  | HLU73 | TGCAACAGCGCGAACGGGAACCCGGCCCCCAAG |
|  | HLU34 | CGTCTAGACCCACTCCAGCCTGGGAGGTCTG |
